# Are *in vivo* selections on the path to extinction?

**DOI:** 10.1111/1751-7915.12490

**Published:** 2017-01-03

**Authors:** José Berenguer, Mario Mencía, Aurelio Hidalgo

**Affiliations:** ^1^ Department of Molecular BiologyUniversidad Autónoma de MadridCenter for Molecular Biology ‘Severo‐Ochoa’ (UAM‐CSIC)Nicolás Cabrera 1Madrid28049Spain

## Abstract

Droplet microfluidics will become a disruptive technology in the field of library screening and replace biological selections if the central dogma of biology and other processes are successfully implemented within microdroplets.

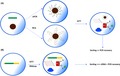

For those of us who work in small laboratories, finding a suitable enzyme for biocatalysis in a metagenomic, random or combinatorial library is always limited not so much by the diversity we can generate, but by how much of that generated diversity we can screen within reasonable economic effort and workforce. Robotics is mostly out of the question for economic reasons, and there is a limit to what a Ph.D. student, postdoc or technician can screen before the task becomes too time‐consuming or worse, too tedious and frustrating. Therefore, we turn to microbial selection assays, which couple the property being evolved to the survival of a host under selective pressure.

There are many advantages to using microbial selections, such as their low cost and high throughput compared with the equivalent screening assays (Packer and Liu, 2015). However, their most relevant property is the provision of the genotype–phenotype linkage required for evolution (Colin *et al*., 2015b; van Vliet *et al*., 2015). The existence of this link ensures that after the phenotype is selected, the genotype will be propagated (in nature) or identified (in laboratory selections). Nature implemented this linkage in an astonishingly simple and elegant fashion: compartmentalization within a living cell.

Unfortunately, the need for living cells in microbial selections entails that certain properties of interest to industry, such as enzyme activity in the presence of organic solvents, activity at high temperatures, enantioselectivity or activity towards unnatural substrates, cannot be straightforwardly implemented, i.e., compatible with the ‘standard’ growth conditions of a microbial host. Nevertheless, a combination of human's creativity and microbial diversity has allowed to turn some phenomena of interest to industry such as protein folding at high temperature (Chautard *et al*., 2007), enantioselectivity (Fernández‐Álvaro *et al*., 2011), interactions at high temperature (Nguyen and Silberg, 2010) into selectable properties. Independently of these clever examples, there are still further problems that hinder the use of microbial selections, such as a possible toxicity of the protein of interest to the host, the capacity of the host to recombine the gene of interest and sometimes the limit to the throughput of the selection imposed by the competence of the host.

These drawbacks have been averted by the replacement of cells with water‐in‐oil droplets, shifting the focus from *in vivo* selections to *in vitro* screenings as demonstrated over the course of the past 20 years by Tawfik and Griffiths (Tawfik and Griffiths, 1998; Griffiths and Tawfik, 2003), Holliger (Ghadessy *et al*., 2001), Abate (Agresti *et al*., 2010), Hollfelder (Kintses *et al*., 2010; Theberge *et al*., 2010; Zinchenko *et al*., 2014; Colin *et al*., 2015b), Hilvert (Obexer *et al*., 2016) and others. In these assays, the genotype–phenotype linkage is achieved compartmentalizing monoclonal DNA and the expressed protein within the same compartment (Fig. 1). In certain droplet screenings, to select antibody modules or binders, a single DNA molecule can be retrieved due to the fact that a covalent link of genotype and phenotype is generated so that the DNA template can be quantitatively captured and retrieved in good yield. However, in screens for enzyme activity, given the fact that a larger amount of DNA is needed to increase both the sensitivity of the method and the recovery of positive clones, methods reported so far make use of high copy number plasmids and whole cells in droplets (Colin *et al*., 2015a; Romero *et al*., 2015) or encapsulate more than one molecule of DNA per droplet, at the cost of monoclonality (Sunami *et al*., 2006). Therefore, the question arises whether to implement *in vitro* screens, we remain bound to use cells, or whether we can replicate the central dogma of biology efficiently enough within an artificial compartment to afford starting from a single DNA molecule per droplet. In our opinion, achieving this goal in a straightforward manner and in good yield will be a disruptive advancement in the field of library screening and will shift the balance towards the use of *in vitro* methods.

**Figure 1:**
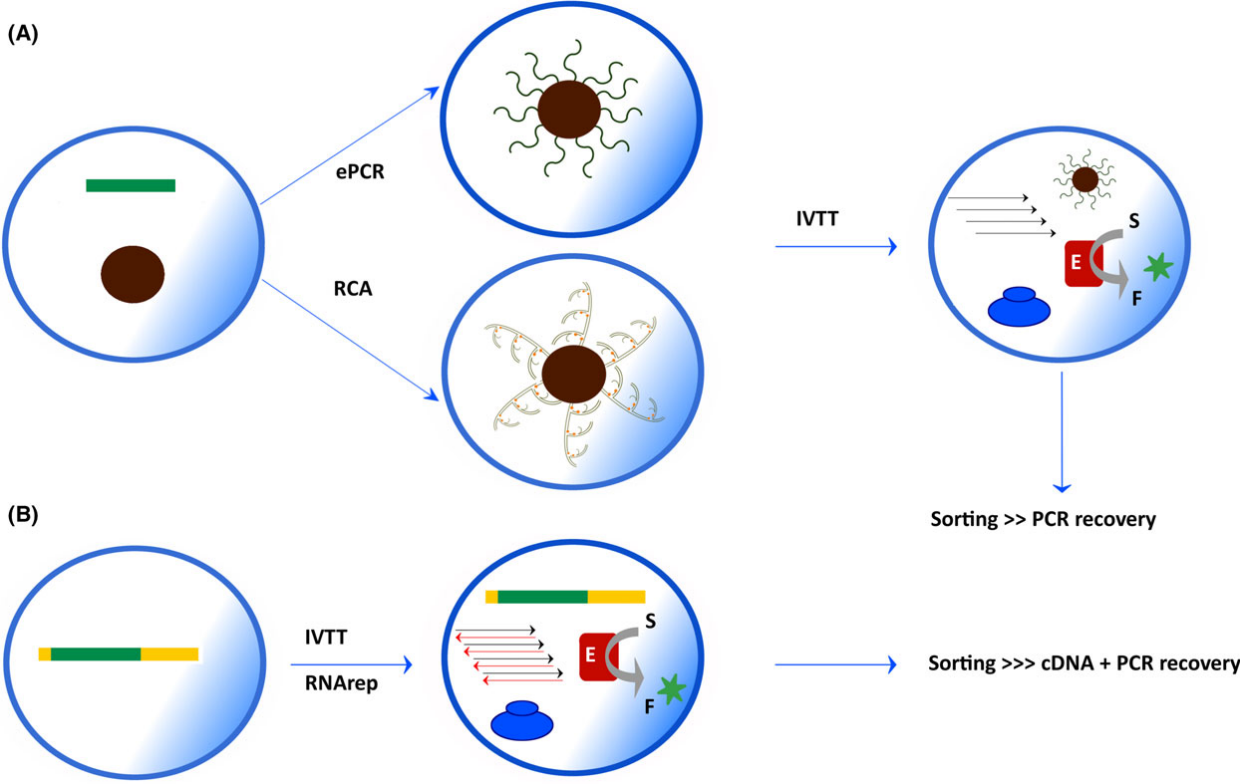
Scheme of future directions for *in vitro* screening. A. A single DNA molecule is encapsulated in microdroplets together with magnetic beads decorated with streptavidin and biotin‐labelled specific primers and the DNA polymerases required either for PCR or for rolling circle amplification. The amplified product is de‐emulsified and re‐emulsified with an *in vitro* transcription and translation (IVTT) system for enzyme production, leading to its detection with a chromogenic substrate (S) that generates a fluorescent product (F*). Positive droplets are sorted and de‐emulsified, and the corresponding coding gene directly recovered from magnetic beads and amplified by PCR. B. A single DNA molecule inserted in phage DNA gene encoding a replicase and flanking replication sequences (i.e. Qβ replicase) is encapsulated directly with an IVTT system. Transcription to mRNA+ from this single DNA copy will suffice to generate the phage replicase, which in turns generates dsRNA copies. Positive RNA strands will be used for translation to generate the enzyme that is detected by fluorescent product production. In this case, sorting of the positive droplets will have to be followed by cDNA generation and PCR for gene product recovery.

To reproduce the central dogma of biology, we will consider first *in vitro* transcription and translation (IVTT). Regarding transcription, the synthesis of RNA should be driven by a single molecule of template DNA and yield sufficient material to detect the translated product. This is usually solved by making use of the strong T7 promoter. The ability to translate RNA into proteins *in vitro* has been long solved by the use of cell‐free extracts from different sources, from the more conventional *Escherichia coli* (Nirenberg and Matthaei, 1961)*,* several *Bacillus* species (Imsande and Caston, 1966; Coleman, 1967; Deutscher *et al*., 1968)*, Pseudomonas* (Broeze *et al*., 1978) to the more unconventional bacterial and archaeal thermophiles (Ohno‐Iwashita *et al*., 1975; Hethke *et al*., 1996). Furthermore, cell‐free expression systems based on pure components have been reported for *E. coli* and *Thermus thermophilus* (Shimizu *et al*., 2001; Zhou *et al*., 2012). However, the low diversity of bacteria from which these IVTT systems have been developed limits the gene signals that may be recognized, which is relevant for functional metagenomics, for instance (Angelov *et al*., 2009).

DNA replication would not be needed if a single molecule of DNA could suffice to drive protein synthesis efficiently enough to detect the product without the need for complex experimental set‐ups (Courtois *et al*., 2008; Okano *et al*., 2012). However, when more widely available flow cytometers are used to screen droplets, the strong template dilution needed to ensure strict monoclonality coupled with limited sensitivity of the technique forces droplet‐based screenings to include a prior and separate template amplification step to achieve a sufficiently strong signal for efficient detection (Fig. 1A). Whereas in prokaryotes, the whole workflow from DNA to protein has evolved to successfully take place within a single compartment*,* an *in vitro* mimic of this workflow requires uncoupling of the different stages, basically replication on one side and transcription–translation in the other. This is due to the incompatibility between IVTT and the two most common DNA amplification techniques: polymerase chain reaction (PCR) and rolling circle amplification (RCA). The incompatibility of PCR is due to the high temperature cycling, while the incompatibility of RCA is attributed to the inhibitory effect of tRNAs and NTPs on replication (Sakatani *et al*., 2015). Possible solutions point to either a two‐step workflow with amplification of encapsulated single DNA molecules on beads (Diamante *et al*., 2013) and their subsequent use as template for IVTT, or compatible blends of isothermal amplification and IVTT.

The coupling of RCA and IVTT has been achieved by elegantly minimizing the inhibition of replication by transcription by decreasing the concentration of NTPs and tRNAs while increasing the concentration of dNTPs. However, the final conditions were made optimal for replication but still may remain suboptimal for translation, given the obtained yield still far from the sensitivity required for enzyme screening (Sakatani *et al*., 2015). A less taxing approach on translation would involve supplementation with components that increase the efficiency of replication, such as the *T. thermophilus* SSB (Inoue *et al*., 2006) or the removal of putatively inhibiting components such as the random hexamer primers.

A beneficial consequence of implementing DNA replication for screening will be the recursivity of DNA replication, i.e., the fact that amplified DNA serves as template for its own amplification, which will enormously facilitate the recovery of the fittest individuals. Nevertheless, recursivity at the DNA level is the most common solution in nature but not the only solution. Recursivity can also take place at the RNA level, using an RNA‐dependent RNA polymerase (Kita *et al*., 2008), which, if compatible with IVTT, may obviate the need for RCA, but it may also complicate the analysis of the selected individuals and introduce bias by imposing a reverse transcription step prior to amplification (Fig. 1B).

Synthetic biologists seek to mimic and eventually redesign life's processes to construct living‐like systems. Some of their discoveries may find use as tools for *in vitro* screening, such as the above‐mentioned reproduction of the central dogma, recursivity or designer regulation of gene expression. On the latter, temporal separation within droplets may be useful in the context of screens with auxiliary enzymes, where an unstable substrate must be generated or an unstable product trapped *in situ,* or when either of them may compromise the stability of the droplet. To implement such one‐pot, time‐delayed assays, we could make use of riboswitches to condition the translation of a messenger RNA to the appearance of a product. As a corollary, the turnover of a substrate into product, neither of which has detectable properties, may be measured as long as the substrate induces the activity of a transcriptional repressor or as acts as ligand of a riboswitch that regulates the translation of a reporter gene. This would be very useful in biocatalysis‐related screens, where substrates of interest are mostly colourless and organic molecules need to be monitored by HPLC or GC.

To sum up our peek into the crystal ball, in our opinion, while synthetic biologists are still far away from their goal in their quest for the designer cell, their partial progress in the form of surrogates for biological processes empowers microfluidic droplet screenings to such an extent where they may be just as flexible and feasible as biological selections and most likely, the face of things to come in the field of high‐throughput screening.
